# Supplementary material for: The HIV-1 vpr R77Q Mutant Induces Apoptosis, G2 Cell Cycle Arrest, and Lower Production of Pro-Inflammatory Cytokines in Human CD4+ T Cells
Source: Viruses. 2024 Oct 21;16(10):1642. doi: 10.3390/v16101642 (PMC11512211; doi:10.3390/v16101642)
Supplement: Supplementary file 1 [file viruses-16-01642-s001.zip › viruses-3156805-supplementary.pdf]

Supplementary Figure 1

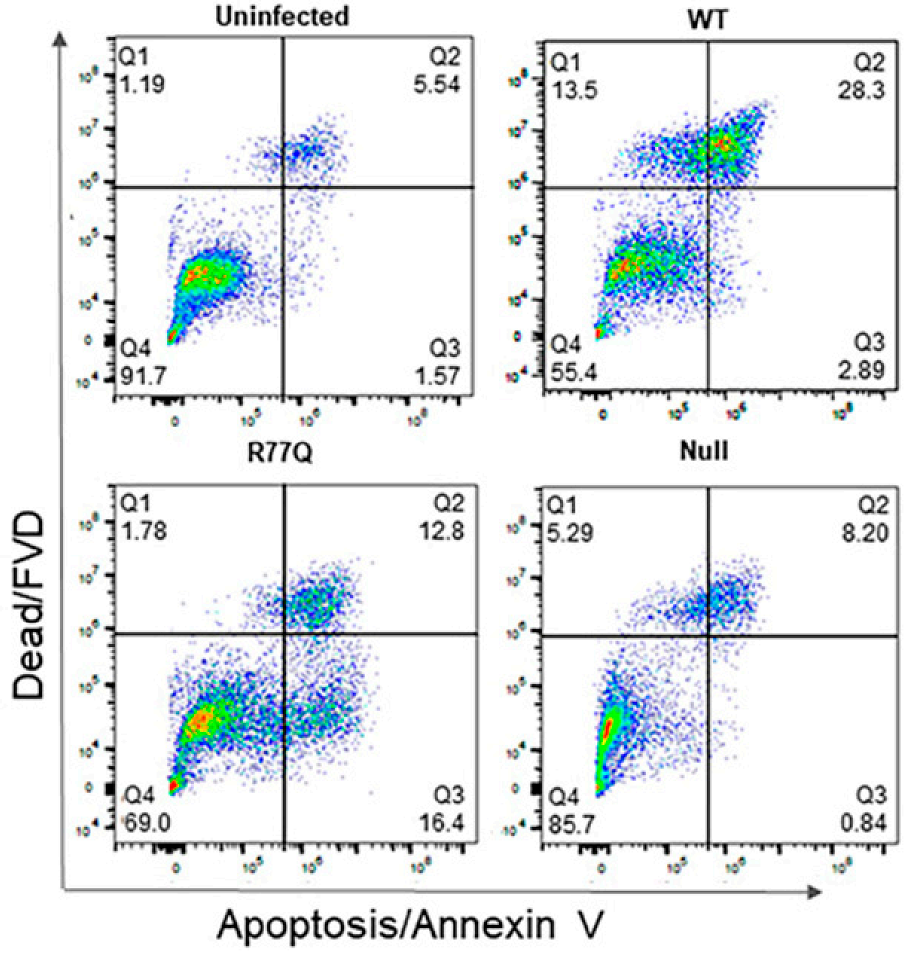

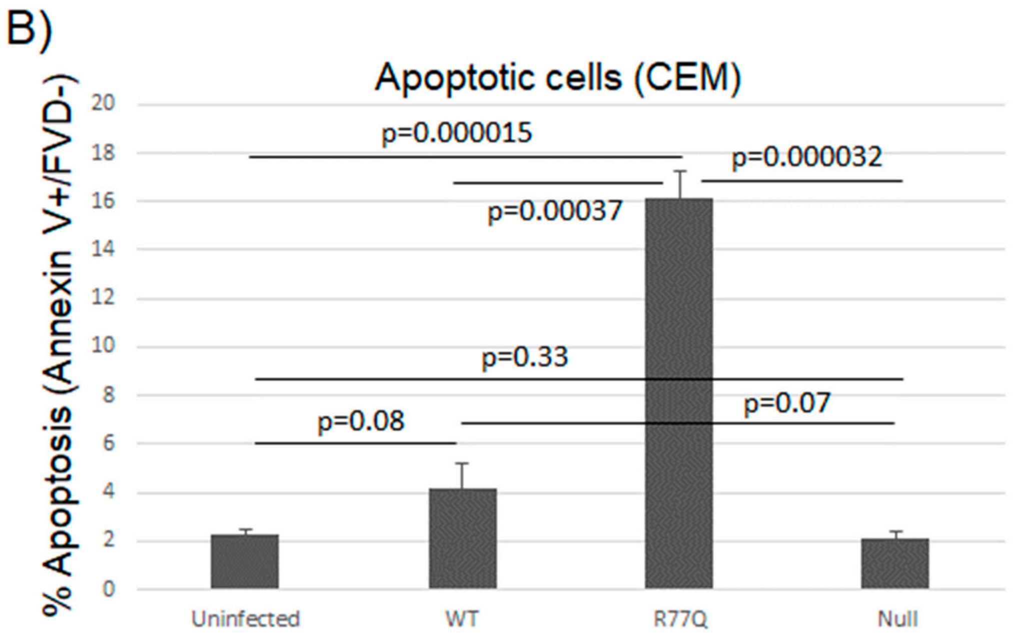

Supplementary Figure 1: The R77Q Vpr mutant triggers apoptosis in CEM cells. WT, R77Q or Null mutants were used to infect HUT78 cells at MOI 0.01. Cell samples were analyzed at 3dpi and panel A shows representative dot plots. Apoptosis and cell death were detected by Annexin V and fixable viability dye (FVD) staining, respectively. Panel B shows means and standard errors for all experiments performed. See supplementary material S6 for statistical analysis. Data are representative of 6 independent experiments.

Supplementary Figure 2

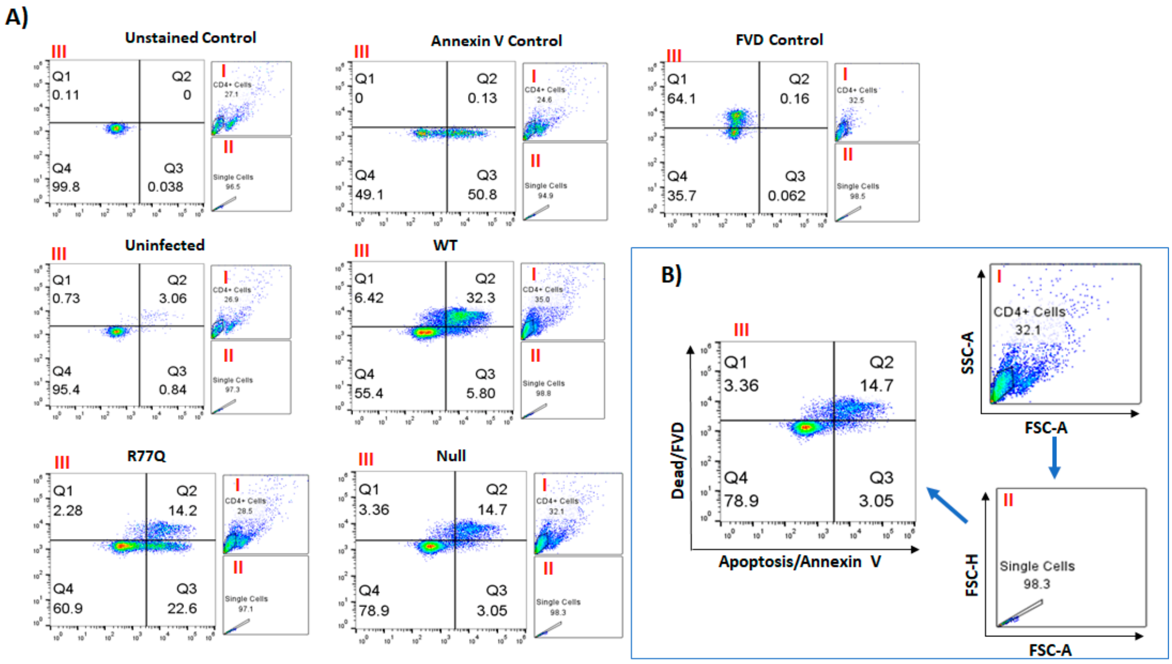

Supplementary Figure 2: CBMC-derived CD4+ T cells become apoptotic after infection with the R77Q mutant (Controls and gating strategy)

Representative dot plots of Annexin V/FVD stains (from patient 2) with ancestry plots. Single positive controls (Annexin V and FVD) and an unstained control were used to guide the gating process. The Annexin V single positive control was created by exposing CD4+ derived CBMCs to 2.5% DMSO for 3 days which created about 50% apoptotic cells after staining for Annexin V. All axis labels for the dot plots and

gating strategy are shown in Sup. Fig. 2B and apply to every dot plot in Sup. Fig. 2A. **A)** Representative dot plots with the associated ancestry plots for patient 2 and the controls used. **B)** Representative dot plot showing axis labels and gating strategy (This applies to every dot plot in **Sup. Fig. 2A**). In brief, the lymphocyte population was gated from CBMCs as shown in panel I. Doublet discrimination was then performed and singlet cells were gated as shown in panel II. Annexin V/FVD gating was then employed as guided by the controls as shown in panel III.
